# Correction to Bratt et al. (2018)

**DOI:** 10.1037/dev0000540

**Published:** 2018-03

In the article “Perceived Age Discrimination Across Age in Europe: From an Ageing Society to a Society for All Ages,” by Christopher Bratt, Dominic Abrams, Hannah J. Swift, Christin-Melanie Vauclair, and Sibila Marques (*Developmental Psychology*, 2018, Vol. 54, No. 1, pp. 167–180. http://dx.doi.org/10.1037/dev0000398), the copyright license has been changed to the Creative Commons CC-BY Attribution License. The online version of this article has been corrected.

